# The influence of low-intensity physiotherapeutic ultrasound on the initial stage of bone healing in rats: an experimental and simulation study

**DOI:** 10.1186/s40349-016-0068-5

**Published:** 2016-10-03

**Authors:** Aldo José Fontes-Pereira, Marcio Amorim, Fernanda Catelani, Daniel Patterson Matusin, Paulo Rosa, Douglas Magno Guimarães, Marco Antônio von Krüger, Wagner Coelho de Albuquerque Pereira

**Affiliations:** 1 Ultrasound Laboratory, Biomedical Engineering Program/COPPE/Federal University of Rio de Janeiro - UFRJ, Rio de Janeiro, Rio de Janeiro Brazil; 2 Laboratory of Morpho-physiopathology, State University of Pará, Belém, Pará Brazil; 3 Military Police Central Hospital of Rio de Janeiro, Rio de Janeiro, Rio de Janeiro Brazil; 4 Laboratory of Epithelial Biology, Department of Periodontics and Oral Medicine, University of Michigan School of Dentistry, Ann Arbor, MI USA

**Keywords:** Physical therapy, Fracture healing, Ultrasonic therapy, Bone

## Abstract

**Background:**

Low-intensity physiotherapeutic ultrasound has been used in physical therapy clinics; however, there remain some scientific issues regarding the bone-healing process. The objective of this study was to investigate the influence of low-intensity physiotherapeutic ultrasound on the initial stage of bone healing in rats.

**Methods:**

Twenty-two male adult rats were assessed quantitatively and qualitatively using radiographic, biochemical, and histological analyses. Numerical simulations were also performed. Fractures in animals in the ultrasound group (*n* = 11) were treated with low-intensity ultrasound (pulsed mode, duty cycle 20 %) for 10 min daily at an intensity of 40 mW/cm^2^ SATA (1.0 MHz) for 10 days. Fractures in animals in the control group (*n* = 11) were not treated.

**Results:**

Alkaline phosphatase levels were non-significantly higher in the ultrasound group than in the control group in the time intervals considered (*t*(13) = 0.440; 95 % confidence interval (CI) −13.79 to 20.82; *p* = 0.67). Between-group serum calcium levels were also not significantly different (*t*(13) = −0.842; 95 % CI −0.48 to 0.21; *p* = 0.42). Finally, there were no significant differences in radiological scores between the two groups (*U* = 118; 95 % CI −1.99 to 1.99; *p* = 0.72). However, the diameter of the newly formed bone tissue was greater and more evident in the ultrasound group.

**Conclusions:**

Thirteen days after fracture, there was no significant between-group differences in bone-healing processes, although the increased alkaline phosphatase levels and diameter of new bone tissue need to be further investigated.

## Background

Even though it is one of the most rigid and resilient substances in the human body, bone tissue is constantly exposed to conditions, such as injury or fracture, that may affect its structural integrity [1, 2]. The occurrence of a fracture triggers a complex process of healing to restore bone mechanics and functional integrity [1, 3]. This process is dynamic and features well-defined stages of repair regulated by a variety of cellular elements and stimulant agents [4]. Sometimes, there can be complications in this process, resulting in retardation of fracture union with the risk of pseudoarthrosis [5] and other consequences, such as long and painful treatment, missed work, reduction in patients’ quality of life and general well-being, and increased public health-care expenditures [6, 7]. Thus, efforts to determine treatments that accelerate the bone consolidation process are justified [2, 8].

Physiotherapy offers several options for treating fractures, including therapeutic ultrasound (TUS), which is normally used in physical therapy clinics [9–11]. According to Wolff’s law, ultrasonic stimulation generates micro-mechanical forces and tension on the fracture site, resulting in accelerated bone formation. It has also been mentioned that the use of low-intensity pulsed ultrasound stimulation (LIPUS) increases bone metabolism [8, 12, 13], resulting in accelerated bone healing by abbreviating inflammation and soft and hard callus formation [14]. Well-documented [8, 12, 14] studies with low-intensity ultrasonic waves have shown evidence of their effects on bone healing. Commercial equipment especially designed to provide low-intensity ultrasound for the purpose of bone healing is normally set at a fixed intensity of 30 mW/cm^2^. However, this equipment costs about 10 times more than does TUS equipment commonly used for general purposes in physical therapy clinics. An investigation of this potential use should include careful steps to enable standardization of the duration and intensity of irradiation required for effective treatment. To date, no conclusive investigation on the evaluation of cellular and biochemical mechanisms triggered by TUS [2, 9] has been reported. The present study analyzed the radiographic, biochemical, and histological effects of TUS at an intensity of 40 mW/cm^2^ in induced fractures of rat tibias with the objective of evaluating the effect of ultrasound intensity provided by common TUS equipment on the initial stage of fracture healing.

## Methods

The study was approved by the Ethics Committee in Research of the Evandro Chagas Institute, Pará, Brazil (protocol n.009/2012) according to the guidelines for the care and use of laboratory animals [15], National Legislation of Animal Vivisection in Force (Federal Law 11,794 of October 8, 2008), and international and national ethical instructions (Laws 6638/79, 9605/98, Decree 24665/34).

### Sample

The sample consisted of 22 male rats (*Rattus norvegicus*, McCoy strain) at least 90 days of age and weighing 325 ± 25 g. Each rat was maintained at a controlled temperature (23 ± 2 °C) in cages measuring 45 × 15 × 30 cm and lined with autoclavable rice straw that was exchanged on alternate days. The animals received water and food ad libitum.

The rats were randomly divided into two experimental groups: a control group (CG), consisting of 11 rats that underwent induced fractures in the middle one third of the right tibia without receiving any treatment, and an ultrasound group (USG), consisting of 11 animals that underwent the same fracture procedure and received low-intensity TUS.

### Fracture induction

Prior to fracture induction, the rats were anesthetized with intraperitoneal ketamine (80 mg/kg) and xylazine (15 mg/kg) solution [2, 8] at a dose of 0.6 mL per 100 g body weight. After sedation, the rats were placed in the lateral decubitus position and then subjected to fracture of the middle one third of the diaphysis of the right tibia of the hind limb, using the equipment previously described [2]. Afterwards, the rats were placed in cages, with a maximum of four rats per cage, and subjected to analgesic therapy during the entire experimental period (200 mg/kg paracetamol dissolved in water). The animals were not immobilized after the fracture [2, 16, 17].

### Treatment

After 24 h, TUS treatment was applied to the fracture gap while the animal was in the lateral decubitus position. The rats were not sedated during treatment. Stationary ultrasound equipment (BIOSET® model SONACEL PLUS, Bioset Industry Electronic Technology Ltda., Rio Claro, SP, Brazil) was used on the fracture site, with a frequency of 1 MHz, intensity of 40 mW/cm^2^ (SATA), pulsed mode, duty cycle 20 %, pulse repetition frequency of 100 Hz, pulse width of 2 ms, and an effective radiating area of 0.79 cm^2^. The equipment was found to meet the IEC 60601-1-2:2010 regulation, according to the calibration made by the Electrical Engineering Department of the Federal University of Pará (Brazil). The coupling material was a commercial gel soluble in water. Treatment was performed for 10 min once per day for five consecutive days, followed by a 2-day period without irradiation. This sequence was repeated for a total of 10 sessions.

### Post-treatment procedures

Once the treatment protocol was complete, the animals fasted for 12 h. Then, they were anesthetized intraperitoneally with hydrochloride ketamine (80 mg/kg) and xylazine (15 mg/kg) at a dose of 0.6 mL per 100 g body weight. While completely sedated, the rats underwent exsanguination by cardiac puncture (~5 mL of blood was collected for biochemical analysis) [2] and then euthanized by decapitation.

Analysis of the bone matrix synthesis biochemical markers was performed using a Labtest kit (Vital Scientific NV, Holliston, MA, USA) with absorbance at 590 nm for measurement of serum alkaline phosphatase level and a laboratory kit (Vital Scientific) with absorbance at 570 nm to determine the concentration of serum calcium. Both analyses were performed using the Vitalab Selectra and Chemistry Analyzer automated system (Vital Scientific).

For radiological evaluation, the rats’ right hind limbs were disarticulated from the hip, fixed in buffered 10 % paraformaldehyde, and subsequently submitted to analysis. Radiography was obtained in the lateral view with the same radiographic technique (40 kV × 2 mA), using an exposure time of 0.6 s, and always at the same distance from the X-ray tube (30 cm). Radiographic analysis was performed by two independent observers blinded to the treatment group who examined the callus formation, the quality of bone union, and bone remodeling, according to the radiographic system score for osseous healing [6, 18, 19]. This radiographic scoring system has three categories (periosteal reaction, quality of bone union, and remodeling) regarding fracture healing. The first two categories are scored from 0 to 3 points and the third category has scores from 0 to 2, so the maximum expected score is 8 (the sum of the maximum score for each category) for complete bone fracture repair (Table 1).

**Table 1:** Radiographic scoring system for fracture healing [6, 18, 19]

Categories	Scores			
	0	1	2	3
Periosteal reaction	None	Mild (<50 %)	Moderate (>50 %)	Full—across the defect
Quality of bone union	No new bone in the fracture line—nonunion	Mild bridge (<50 %)	Moderate bridge (>50 %)	Full bone bridge union
Remodeling	No remodeling	Mild remodeling (<50 %)	Full remodeling cortex	–

Finally, the right hind limb was dissected until the tibia was totally exposed and immersed for 36 h in a descaling solution of ethylenediaminetetraacetic acid (EDTA) at 5 %. Then, histological slides were prepared with the histotechnical procedure and a microtome (Leica Microsystems, Wetzlar, HE, DE). Sections of 5-mm-thick tissue were obtained from the region of the bone callus and stained with hematoxylin-eosin [20]. Qualitative analysis of the blades by evaluation of bone formation from estimation of the thickness of the newly formed tissue was made with an optical microscope (Carl Zeiss Microscopy LLC, Thornwood, NY, USA) coupled to a video camera, the AxioCam HRC (Carl Zeiss Microscopy LLC, Thornwood, NY).

### Simulation configuration

**Fig. 1:**
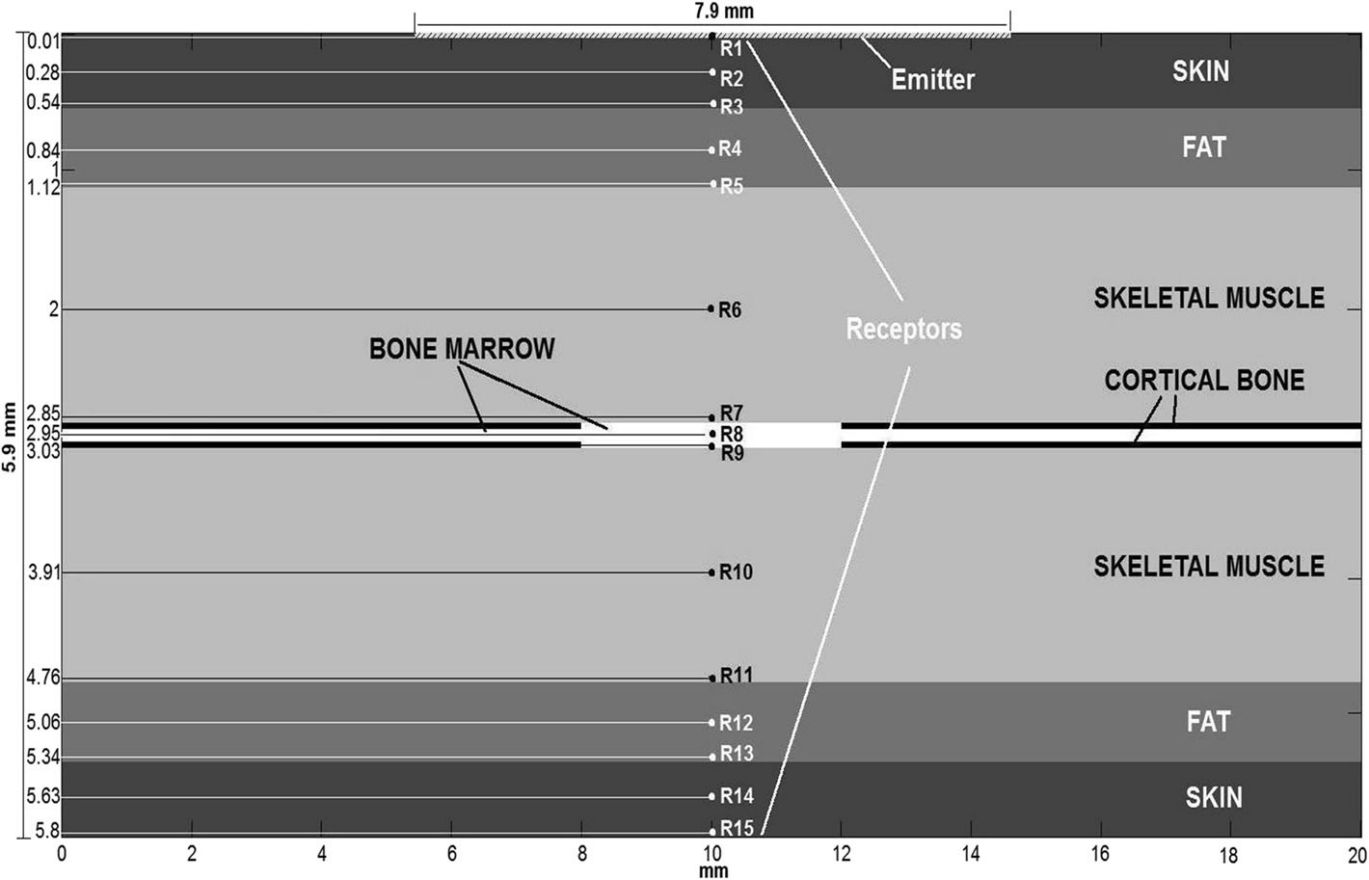
Diagram of the numerical model configuration

Numerical, two-dimensional simulations of wave propagation were performed with SimSonic software developed at the Laboratoire d’Imagerie Paramétrique (CNRS, University Paris 6, Paris, France), employing the method of finite-difference time domain (FDTD) with elastodynamic equations (Fig. 1) [21].

A numerical model with a spatial resolution of 0.01 mm represented the region of the fracture, and it consisted of two cortical plates with bone marrow inside surrounded above and below by muscle, fat, and skin. Interruption of the cortical bone represented the fracture gap. The thickness of the cortical layers and bone marrow was 0.04 and 0.1 mm, respectively, and the fracture gap was 4 mm. The thickness of the muscle, fat, and skin was 1.73, 0.58, and 0.55 mm, respectively. These values are averages of measurements obtained from the radiography of the animals used in the experiments.

In the two-dimensional numerical model, a line source measuring 10 mm placed on the skin layer’s upper surface generated a longitudinal pulsed wave of 1 MHz with a duration of 3 μs. Fifteen receivers (R1–R15) positioned along the propagation axis recorded pressure during 20 μs starting from just before the pulse generation. The receivers R2, R4, R6, R8, R10, R12, and R14 were located in the middle of each layer, while the receivers R3, R5, R7, R9, R13, and R15 were located over the interface between layers (1 pixel apart). Perfect matching between layers (PML) was assumed, and absorption was disregarded.

The elastic constants (C11, C22, C33, and C12) and densities (Table 2) obtained from the literature [21] were used to model the isotropic mechanical responses of each material. The longitudinal velocity of isotropic materials and the calculated acoustic impedance were within the range of values reported in the literature [22].

**Table 2:** Mechanical properties of tissues [21]

Tissue	*E* (GPa)	*σ*	*ρ* (kg/m^3^)	*C*_l_ (m/s)	*Z* (kg/m^2^ s)
Skin	0.000035	0.499998	1050	1666.67	17.50
Fat	0.000035	0.4999971	940	1462.84	13.75
Muscle	0.000012	0.4999993	1040	1657.48	17.24
Bone marrow	0.002	0.49985	1020	1476.32	15.06
Cortical bone	15	0.37	1970	3669.74	72.29

*E* Young’s modulus, *σ* Poisson’s ratio, *ρ* density, *C*_*l*_ longitudinal velocity, *Z* acoustic impedance

### Parameters evaluated

The parameters used in the analysis were time-of-flight of the first arriving signal (TOF_FAS_), sound pressure level (SPL), and amplitude root mean square (RMS) [23]. The TOF_FAS_ evaluates the duration of the ultrasound wave propagation leaving the emitter transducer until its arrival at the corresponding receiving transducer, responding to impedance differences in the propagation medium. The TOF_FAS_ was obtained by interpolating the five amplitude points by a parabolic signal adjustment of each receiver from a given threshold.

The SPL and RMS were used to evaluate ultrasound wave amplitudes, providing, respectively, attenuation and energy of the signal of each receiver. While the SPL was calculated based on the peak of the wave (Eq. 1), the RMS (Eq. 2) used a temporal window of 10.9 μs from the first arriving signal (FAS), as follows:

1 $$ \mathrm{S}\mathrm{P}\mathrm{L}=20\cdot { \log}_{10}\left(\frac{A_{\mathrm{R}\mathrm{k}}}{A_{\mathrm{R}1}}\right), $$

where *A*_Rk_ is the peak amplitude of the signal of receivers R2 to R15 (*k*) and *A*_R1_ corresponds to the amplitude of the reference signal (receiver R1).

2 $$ \mathrm{R}\mathrm{M}\mathrm{S}=\sqrt{\frac{{\displaystyle {\sum}_{k=1}^{k=N}{A_{\mathrm{Rk}}}^2}}{N},} $$

where *A*_Rk_ corresponds to the signal amplitude of each receiver (*k* = 1–15) divided by the total number of signals (*N*).

### Statistical analysis

Power tables for Cohen’s *d* effect size were used to calculate the sample size: Cohen’s *d* = 1.2 two-tailed, *α* = 0.05, and power of 0.8 [24]. Data normality was examined using the Kolmogorov-Smirnov test. Student’s *t* test (*t*) was used to evaluate differences in biochemical markers, and the Mann-Whitney *U* test (*U*) was used to evaluate differences in radiographic scores between the groups. The inter-observer agreement was calculated using the kappa coefficient (*K*). Statistical analysis was performed using SPSS (version 20.0, IBM Corporation, Armonk, NY, USA). The level of significance was *α* = 0.05, with a confidence interval of 95 %.

## Results

The USG presented higher levels of alkaline phosphatase (86.38 ± 18.94 U/L) than did the CG (82.86 ± 10.03 U/L) for the time interval considered, but with no statistical significance (*t*(13) = 0.440; 95 % CI −13.79 to 20.82; *p* = 0.67). Serum calcium levels also were not significantly different (*t*(13) = −0.842; 95 % CI −0.48 to 0.21; *p* = 0.42); however, the CG had higher levels of serum calcium (10.04 ± 0.26 mg/dL) than did the USG (9.90 ± 0.35 mg/dL). Seven samples were discarded because of coagulation.

**Fig. 2:**
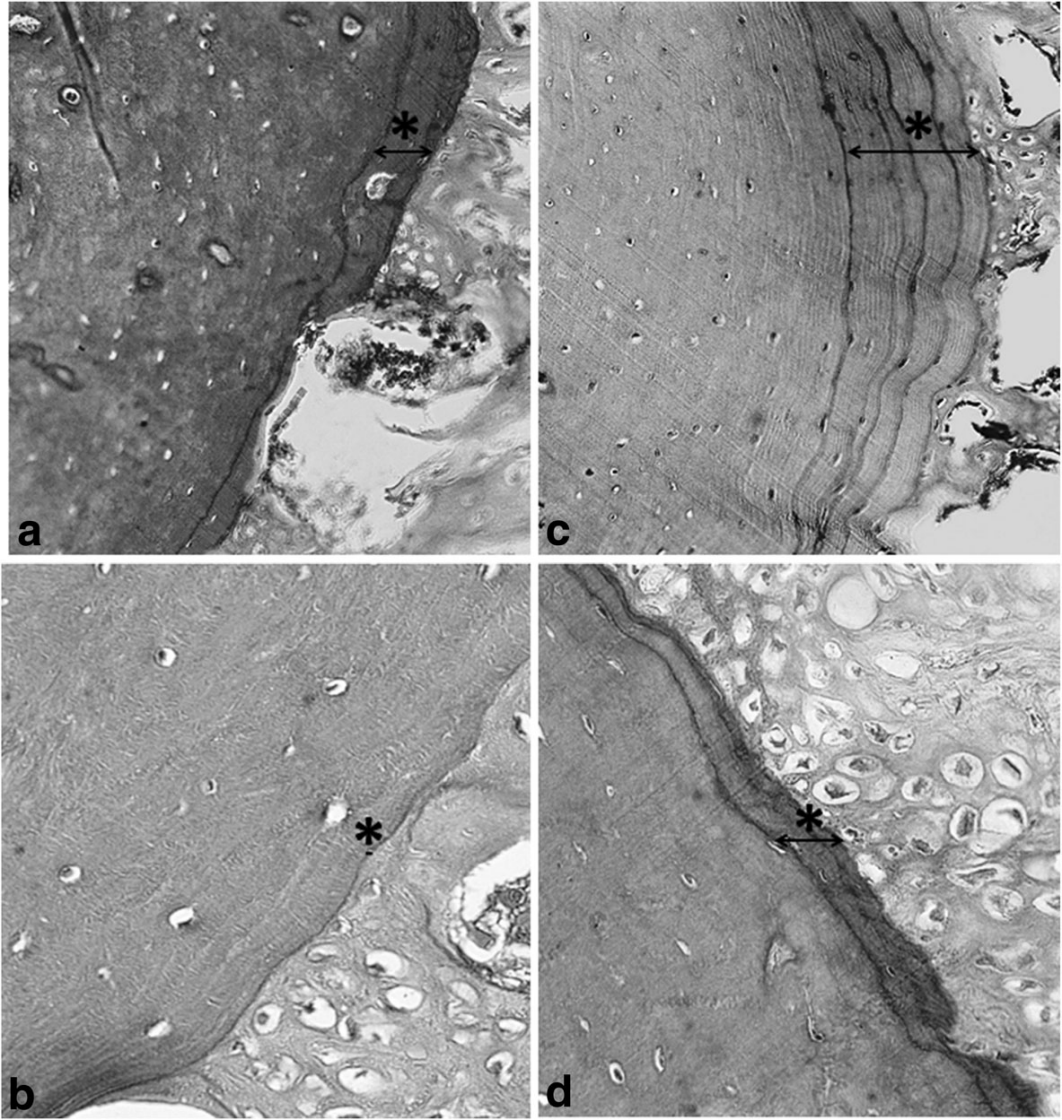
Formation of an immature bone in the control group (CG) (**a** hematoxylin and eosin (H&E) ×20 and **b** H&E ×40) and the ultrasound group (USG) (**c** H&E ×20 and **d** H&E ×40) was similar. The diameter of the newly formed bone tissue (*asterisk*) was greater and more evident in the USG

The qualitative histological analysis revealed the formation of immature bone in both groups. However, the diameter of the newly formed bone tissue was greater and more evident in the USG (Fig. 2).

**Fig. 3:**
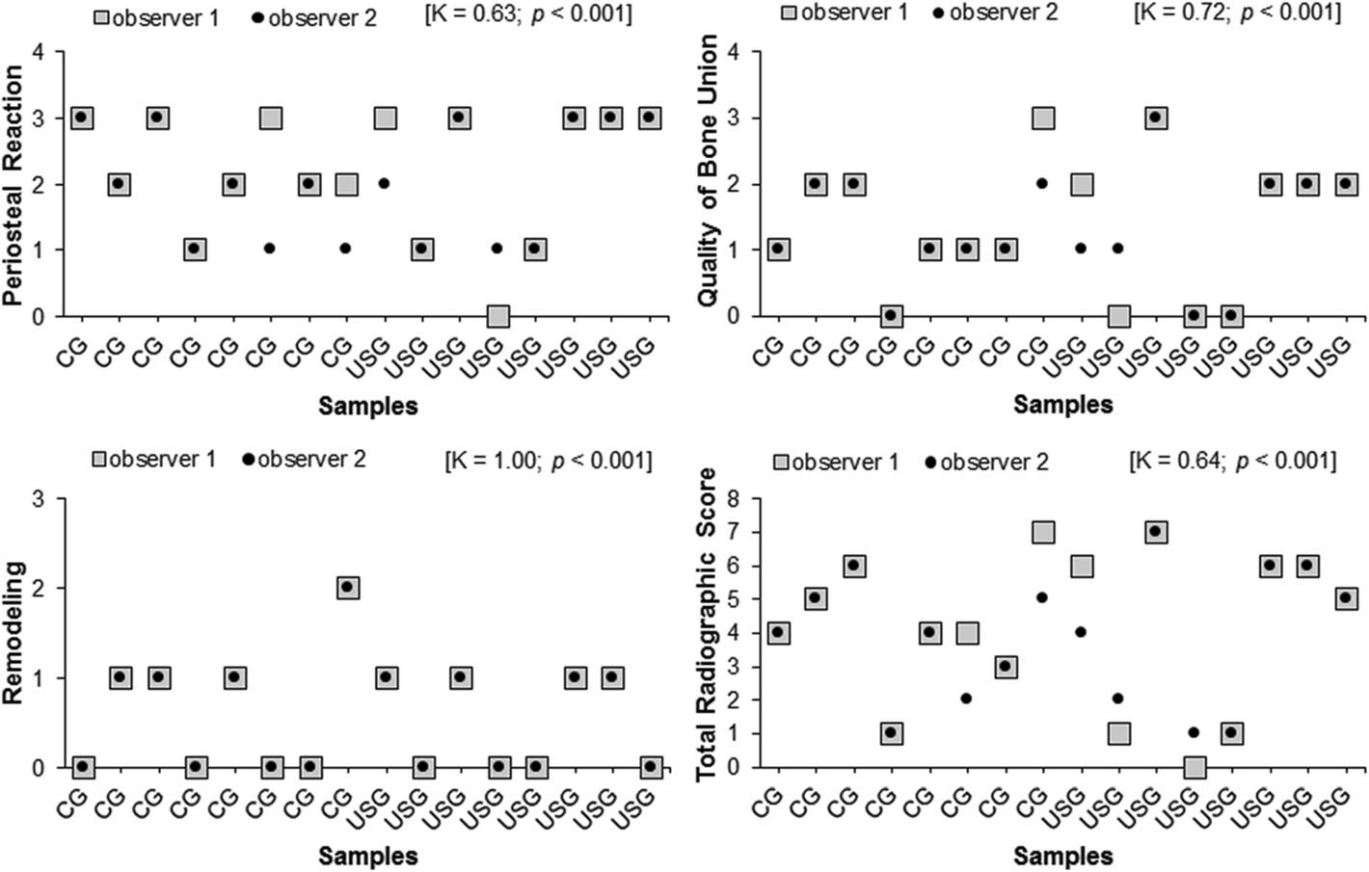
Radiographic scoring system for fracture healing and categories (periosteal reaction, quality of bone union, and remodeling)

The inter-observer reliability (*r*) for the total radiographic score was *K* = 0.64 (*p* < 0.001) and for the three categories periosteal reaction, quality of bone union, and remodeling was *K* = 0.63 (*p* < 0.001), *K* = 0.72 (*p* < 0.001), and *K* = 1.00 (*p* < 0.001), respectively. The scoring system for radiographic fracture healing showed no significant difference between the groups (*U* = 118; 95 % CI −1.99 to 1.99; *p* = 0.72) (Fig. 3).

**Fig. 4:**
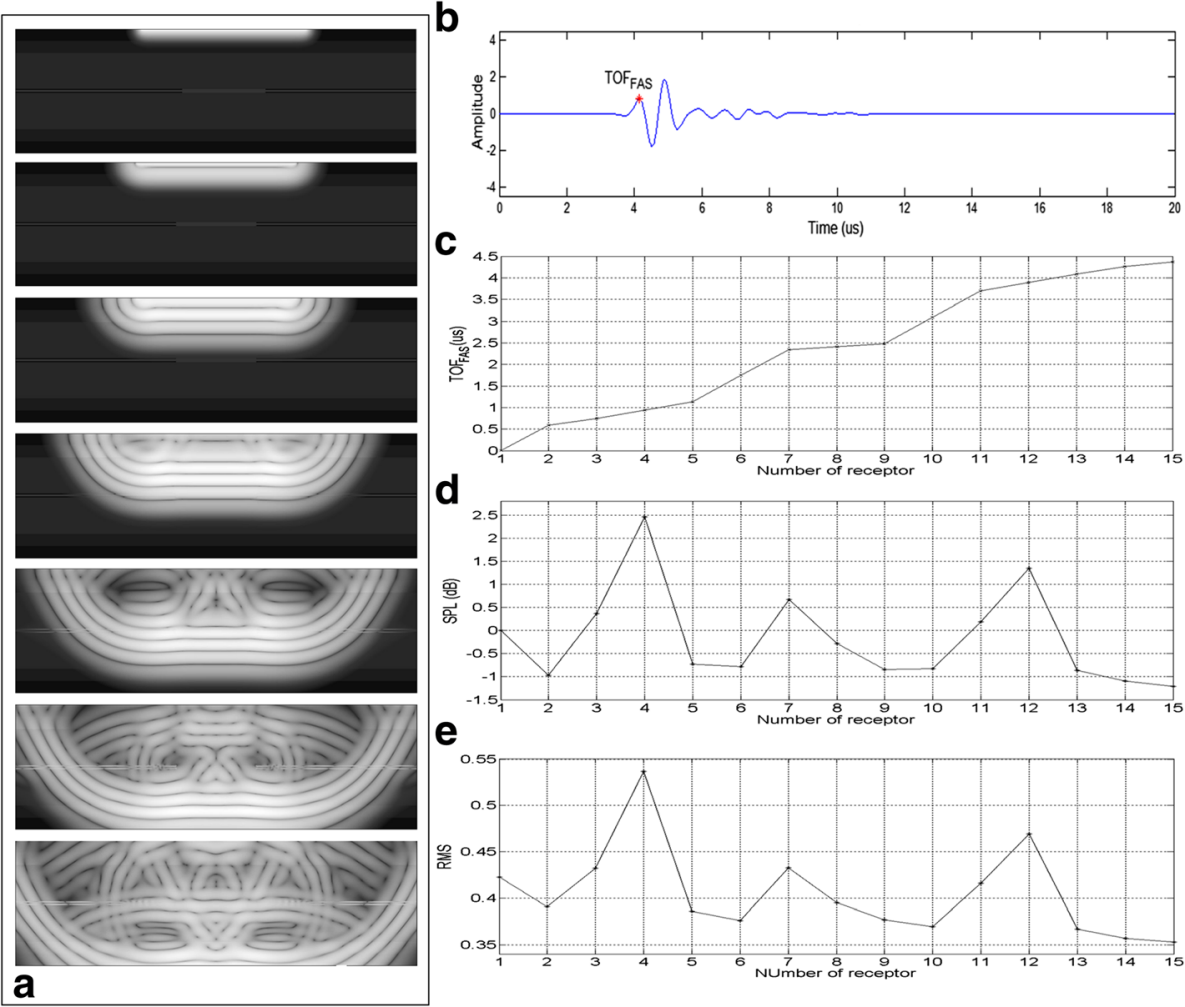
**a** Wave propagation along the tissue. **b** Signal of receiver R8 positioned in the center of the fracture gap showing the time-of-flight of the first arriving signal (TOF_FAS_). **c** TOF_FAS_ of receivers R1 to R15. **d** Sound pressure level (SPL) of receivers R1 to R15. **e** Root mean square (RMS) of receivers R1 to 15

Figure 4a shows wave propagation along the tissue, Fig. 4b shows an example of a typical signal detected by the receivers, and Fig. 4c presents the TOF_FAS_, which increased with the depth of the receivers and the thickness of tissue. It should be noted that between the signal of receiver R1 (positioned at the top of the model) and R2 (0.28 mm from the top of the model), there is a computational adjustment mechanism for TOF_FAS_ prediction, so this parameter is considered only from receiver R2. Figure 4d, e shows the attenuation and signal energy of each receiver, respectively.

## Discussion

The use of conventional TUS for fracture treatment could mean a reduction in treatment costs for bone fractures, therefore making this therapeutic modality more accessible to the general population. Thus, this work was intended to elucidate the effects of TUS on the bone-healing process. The animal model *R. norvegicus* (McCoy strain) was used, as it has pathophysiological and biomechanical properties similar to those of the human bone [11]. A closed-fracture model was chosen to reduce the risk of infection, which would alter the consolidation process [2]. Several studies [2, 17] did not have success using fracture stabilization methods in rats, whether it was by invasive treatments, such as Kirschner wires, or noninvasive treatments, such as the use of a plaster splint. Several complications arose, namely bone fractures in other regions of the limb, compartment syndrome, and infection at the site of invasive assets. Thus, in the present study, none of the fractures was immobilized.

The method of treatment adopted for this study follows the usual human physical therapy treatment protocol. According to Einhorn [25], between 10 and 16 days, it is possible to identify four healing stages. Our protocol lasted 13 days, so we can assume that the healing process was occurring and that our results indicate that the ultrasonic dose we used was not able to accelerate this process.

Biochemical analysis was performed using the indices of alkaline phosphatase and serum calcium. These markers were used to evaluate the process of bone formation [13, 16, 26] once calcium became a component of the bone matrix and alkaline phosphatase activity was noted in osteoblastic formation [26, 27]. The results of this biochemical analysis were not statistically significant, although ultrasound did promote increased alkaline phosphatase activity in the treated versus the untreated rats. These results were similar to those in a previous study [2] that tested TUS pulsing (0.2 W/cm^2^) in rats with bone fractures after 5 weeks of treatment. Leung et al. [28], using LIPUS equipment designed for bone healing, showed that treatment for 20 min per day at 30 mW/cm^2^ increased levels of alkaline phosphatase. Guerino et al. [29] suggested that an increased level of alkaline phosphatase seen with TUS is possibly associated with increased cell proliferation and mineralization.

Histological analysis showed that animals treated with TUS had the thickest bone formation, suggesting that TUS influenced the consolidation of bone tissue. A similar result was found by Oliveira et al. [30] when LIPUS equipment was used at an intensity of 30 mW/cm^2^. However, our findings are not yet conclusive regarding bone-healing acceleration. Perhaps TUS can influence bone formation, but at least for the dose that we applied, no statistically significant difference was found.

The qualitative radiological analysis showed a greater volume of bone callus in animals in the USG. Thus, as in the study of Kumagai et al. [8], radiological evaluation showed that the area of hard callus was significantly higher in the LIPUS-treated animals than in the animals in the CG. However, the scoring system for radiographic fracture healing showed no significant difference between the groups. These findings support the use of quantitative measures (e.g., quantitative ultrasound, bone densitometry, quantitative computed tomography) that are often overlooked in traumatology studies. Thus, these tools can be used to minimize the subjectivity of evaluators and to show real statistical differences between therapies.

Simulation analysis showed that TOF_FAS_ values were consistent with the localization of receivers in the numerical model so that the highest values were observed with a larger distance between receivers or when they were farther from the emitter. The arrangement of the receivers, when chosen with respect to the thickness of each tissue, showed that the interior of the fracture responded to the smallest change in TOF_FAS_ (receivers R7–R9) (i.e., the wave propagates faster inside the fracture). This fact should be taken into account in case therapy depends on the propagation time of ultrasound inside a given region.

Catelani et al. [23], using SimSonic software (CNRS, University Paris 6, Paris, France), found that the interior regions of fractures near the cortical bone-bone marrow interface showed greater reduction of TOF_FAS_ values with respect to receivers located in the center of the fracture (due to the formation of lateral waves with velocity compatible with the cortical bone). This could account in part for the mechanisms involved in fracture healing stimulated by LIPUS. Additionally, in this study, it was possible to note the reduction of TOF_FAS_ through the interior of the fracture.

The results of the SPL and RMS analyses showed similar behavior between values for different receivers. The highest concentration of energy was observed in receivers near the skin-fat, fat-muscle, and bone marrow-muscle interfaces. The receivers R4, R7, and R12 excelled in energy concentration, followed by the adjacent receivers R3, R8, and R11. The impedance mismatch led to the reflection phenomenon of ultrasound waves at the interfaces, which may explain the higher concentration of energy in the soft tissues and attenuation of the ultrasound wave in the center of the model and deeper regions, where the greatest attenuation would be expected.

The intensity of LIPUS proposed in the literature (30 mW/cm^2^) seems to provide a good stimulus for accelerating the bone-healing process. This intensity would not be directly proportional to a higher concentration of energy at the fracture site, however, given that the intensity of the ultrasound used in this study was approximately 33 % higher (40 mW/cm^2^) and it did not change the duration for bone repair. As the concentration of power and local induction heating are considered harmful to bone healing, factors such as the rapid passage of ultrasound through fracture [23], which is associated with low-intensity TUS, are closely related with the acceleration of consolidation. Conversely, higher intensities would increase the risk of local heating, hindering this process.

The ultrasound equipment commonly found in physical therapy clinics may influence the bone-healing process according to anecdotal evidence from books, blogs, and practitioners, but this claim has little scientific basis [9].

## Conclusions

In this study, we could not find evidence that TUS influences the bone-healing process. On the other hand, some aspects of the procedure need to be clarified (e.g., ultrasonic intensity, duration of treatment) with respect to changes in levels of alkaline phosphatase and the diameter of new bone formation observed in this study. We propose that there is an optimal range for accelerating bone healing, around 30 mW/cm^2^ of ultrasonic intensity, so to use TUS, it would be necessary to use attenuators. Thus, additional studies of different parameters at different stages of bone healing are needed to clarify the interaction between TUS and biological tissue. The present results suggest that TUS in the dose we used is not recommended for clinical use.

## Abbreviation

FAS: First arriving signal
FDTD: Finite-difference time domain
LIPUS: Low-intensity pulsed ultrasound stimulation
RMS: Root mean square
SPL: Sound pressure level
TOF_FAS_: Time-of-flight of the first arriving signal
TUS: Therapeutic ultrasound
